# Stabilometry in Relation to Hip and Knee Muscle Force in Children with Surgically Treated Unilateral Slipped Capital Femoral Epiphysis

**DOI:** 10.3390/children11101186

**Published:** 2024-09-28

**Authors:** Marius Negru, Anca Raluca Dinu, Elena Amaricai, Liliana Catan, Andrei Daniel Bolovan, Adrian Emil Lazarescu, Corina Maria Stanciulescu, Eugen Sorin Boia, Calin Marius Popoiu

**Affiliations:** 1 Doctoral School, “Victor Babes” University of Medicine and Pharmacy, 300041 Timisoara, Romania; marius.negru@umft.ro (M.N.); andrei.bolovan@umft.ro (A.D.B.); 2 Department of Rehabilitation, Physical Medicine and Rheumatology, Faculty of Medicine, “Victor Babes” University of Medicine and Pharmacy, 300041 Timisoara, Romania; amaricai.elena@umft.ro (E.A.); catan.liliana@umft.ro (L.C.); 3 Research Center for Assessment of Human Motion, Functionality and Disability, “Victor Babes” University of Medicine and Pharmacy, 300041 Timisoara, Romania; 4 Department of Anatomy, Faculty of Medicine, “Victor Babes” University of Medicine and Pharmacy, 300041 Timisoara, Romania; lazarescu.adrian@umft.ro; 5 2nd Clinic of Orthopaedics and Traumatology, Timisoara Emergency County Hospital, 300723 Timisoara, Romania; 6 Teodor Sora Research Centre, Department of Orthopaedics and Traumatology, “Victor Babes” University of Medicine and Pharmacy, 300041 Timisoara, Romania; 7 Department of Pediatric Surgery, “Victor Babes” University of Medicine and Pharmacy, 300041 Timisoara, Romania; stanciulescu.maria@umft.ro (C.M.S.); boia.eugen@umft.ro (E.S.B.); mcpopoiu@umft.ro (C.M.P.)

**Keywords:** slipped capital femoral epiphysis, stabilometry, muscle force, postural balance

## Abstract

Background/Objectives: The main aim of our study was to analyze the stabilometric parameters in relation to hip and knee muscle force in children with unilateral slipped capital femoral epiphysis (SCFE) who had undergone surgical treatment. Another objective was to compare the stabilometry in three testing situations (eyes open, eyes closed, and head retroflexed). Methods: In total, 26 patients with unilateral right SCFE treated via in situ fixation with one percutaneous screw performed stabilometry assessments under three different situations (with their eyes open, with their eyes closed, and with their head retroflexed) and isometric muscle force assessment of the bilateral hip flexors, extensors, abductors and adductors and bilateral knee flexors and extensors. Results: No significant differences between the right side (affected hip) and left side (non-affected hip) were recorded for all of the tested muscle groups. We found significant negative correlations between the 90% confidence ellipse area (eyes open condition) and left knee extensors (*p* = 0.028), right knee flexors (*p* = 0.041), and left knee flexors (*p* = 0.02), respectively. When performing the comparison between the eyes open and eyes closed situations, there were significant differences in CoP path length (*p* < 0.0001) and maximum CoP speed (*p* = 0.048); the parameters increased in the eyes closed situation. Conclusions: Better postural stability is acquired when assessed with eyes open or with the head retroflexed in contrast with eyes closed testing.

## 1. Introduction

Slipped capital femoral epiphysis (SCFE) is a common hip disorder that appears in infancy and adolescence. Usually, this is diagnosed in overweight adolescent boys with symptoms such as pain in the groin, thigh, or knee as well as limping. During hip examinations, limited internal rotation and flexion are observed, along with decreased range of motion. A frog-leg lateral pelvis radiograph is necessary to confirm the diagnosis [1].

Obesity is the most significant risk factor for the development of SCFE. Growth hormone use, hypothyroidism, and vitamin D deficiency are also risk factors. The study by Zusman et al. demonstrates the public health implications of obesity and comorbid conditions in SCFE [2]. As in other pediatric hip pathologies, the identification of risk factors and an early diagnosis are essential for the optimal management of this population [3].

The abductor muscle is the muscle most affected by this pathology. Abductor muscle lengths were measured in patients using two methods of surgical correction and in both situations, there were some abnormalities. The abductor lengths decreased in both situations, but there were fewer differences in abductor lengths after femoral neck base osteotomies than after subtrochanteric osteotomies [4].

In patients with unilateral SCFE, hip muscle atrophy is very frequent. Gao et al. [5] investigated the relationship between hip muscle cross-sectional area and unilateral SCFE using magnetic resonance imaging. In patients with unilateral SCFE, hip muscle atrophy is associated with SCFE severity. Good muscle function and maintaining hip muscle morphology bring benefits to the clinical performance of patients with unilateral SCFE. Sangeux et al. [6] reported a change from adduction to abduction of the injured hip in comparison to a normal gait.

Stabilometry measures how much a person sways while standing by using a force platform. This method analyzes the changing center of pressure (CoP) coordinates over time [7]. Nagymate and Kiss studied the correlation and variance analysis of CoP parameters in various standing conditions. They suggested that time–distance parameters are independent CoP parameters sensitive enough to highlight differences in different standing conditions [8]. Postural balance is crucial for various activities, including daily tasks and sports, as it involves keeping the center of gravity over the support base [9]. Keeping a vertical stance is a complex task; it depends on factors related to the vestibular, somatosensory, and visual systems and motor abilities [10].

The main aim of our study was to analyze stabilometric parameters in relation to hip and knee muscle force in children with unilateral SCFE who underwent surgical treatment. We chose to assess the hip and knee muscle force as these are commonly decreased (for both the affected and healthy side) in an orthopedic pathology of the hip joint. Another study objective was to compare the stabilometry in three testing situations (eyes open, eyes closed, and head retroflexed). We hypothesized that the muscle force of the healthy lower limb has higher values in comparison to the affected one. Considering postural balance, we envisaged that there would be differences between the testing conditions, with decreased stability when assessed with eyes closed.

## 2. Materials and Methods

### 2.1. Participants

The current study took place between October 2023 and February 2024. The selection of children was made from the patients treated at the Pediatric Surgery department of “Louis Turcanu” Emergency Children’s Hospital Timisoara, Romania. The inclusion criteria were: children with unilateral SCFE of the dominant lower limb that required surgical intervention, namely in situ fixation with one percutaneous screw, at least 3 months after surgical intervention. The dominant lower limb was determined as the lower limb the subject chooses for kicking [11].

The exclusion criteria were the following: secondary avascular necrosis of the femoral head, secondary chondrolysis of the hip, musculoskeletal disorders except SCFE, neurologic pathology, vestibular or visual disturbances, diseases that can weaken motor performance, and children under 5 years. Two children were excluded (one suffering from cerebral palsy and one with a recent ankle sprain).

We enrolled 26 patients in the study. The minimum sample size was 20 subjects. The sample size was calculated using G*Power (Heinrich-Heine-Universität, Düsseldorf, Germany); the significance level was 0.05, with a power of 0.95 and an effect size of 0.8 [12].

We collected the following patients’ features: age, height, weight, and body mass index (Table 1). All participants were right-leg dominant.

Participation in this study was voluntary; all children’s parents signed an informed consent form. This study was carried out in agreement with the Declaration of Helsinki. 

### 2.2. Assessment

#### 2.2.1. Stabilometry Assessment

Stabilometry was determined using the PoData system (Chinesport, Udine, Italy) [13]. The participants were assessed barefoot (keeping their natural feet position) in a standing position, without moving or talking, for 20 s. The test was not considered valid if the subject moved an arm, lifted a foot, fell out of position, moved their head, or talked.

CoP path length, 90% confidence ellipse area, and the maximum CoP speed were the evaluated time distance stabilometry data [14]. The CoP path length is the length (measured in millimeters) of the participant’s center of gravity shift during the investigation. The confidence ellipse area is the area (calculated in mm^2^) of the ellipse that contains all of the center-of-gravity points computed and transferred on a system of Cartesian axes with a confidence level of 90%. The maximum CoP speed is the average center-of-gravity shifting maximum speed in millimeters per second.

**Figure 1:**
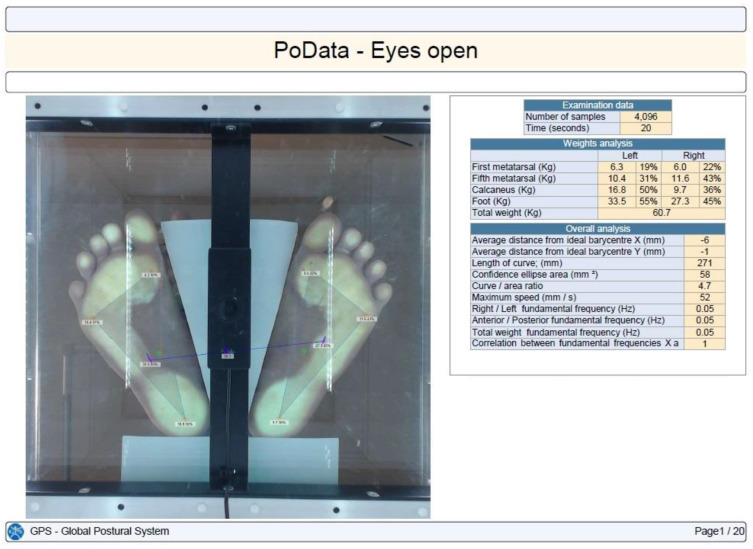
Plantar pressure in a healthy control with eyes open.

**Figure 2:**
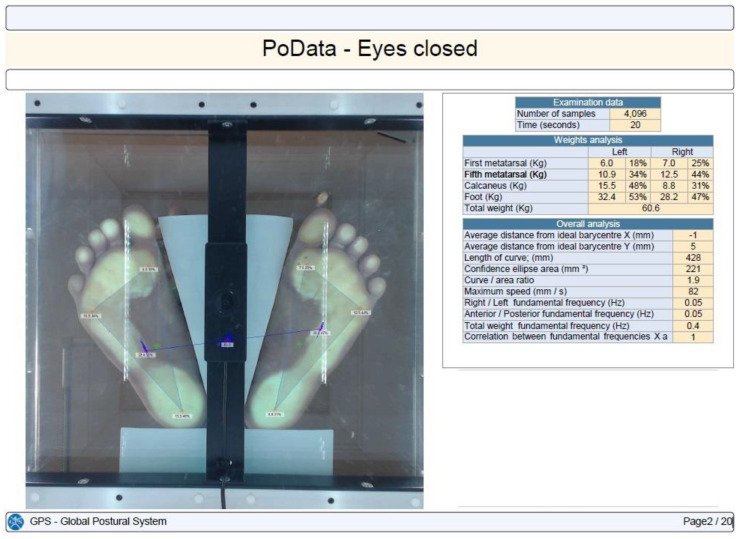
Plantar pressure in a healthy control with eyes closed.

**Figure 3:**
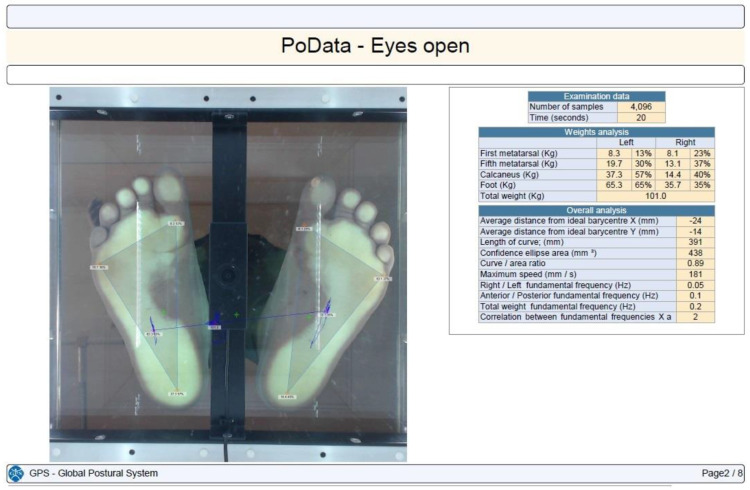
Plantar pressure in an operated SCFE with eyes open.

**Figure 4:**
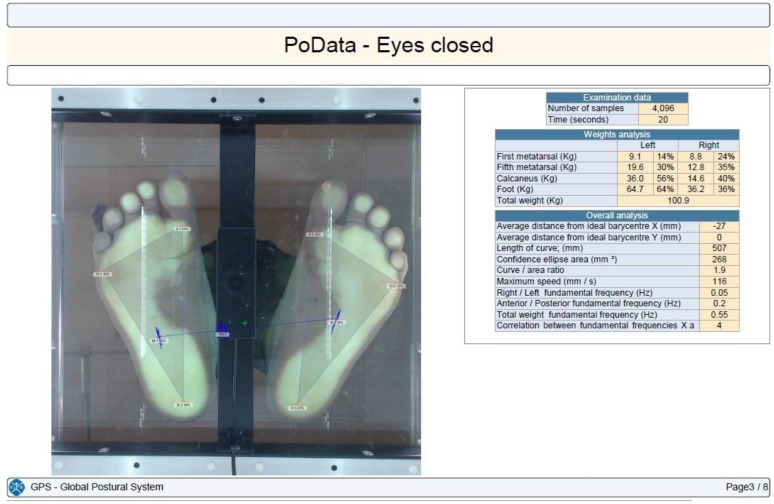
Plantar pressure in an operated SCFE with eyes closed.

Each subject was tested in three different situations: eyes open, eyes closed, and with their head retroflexed. The participants maintained the same foot position between the testing conditions (Figure 1, Figure 2, Figure 3 and Figure 4).

#### 2.2.2. Isometric Muscle Force Assessment

Isometric muscle force was measured using a MicroFet 2 dynamometer (Hoggan Health Industries, Draper, MA, USA) [15].

**Figure 5:**
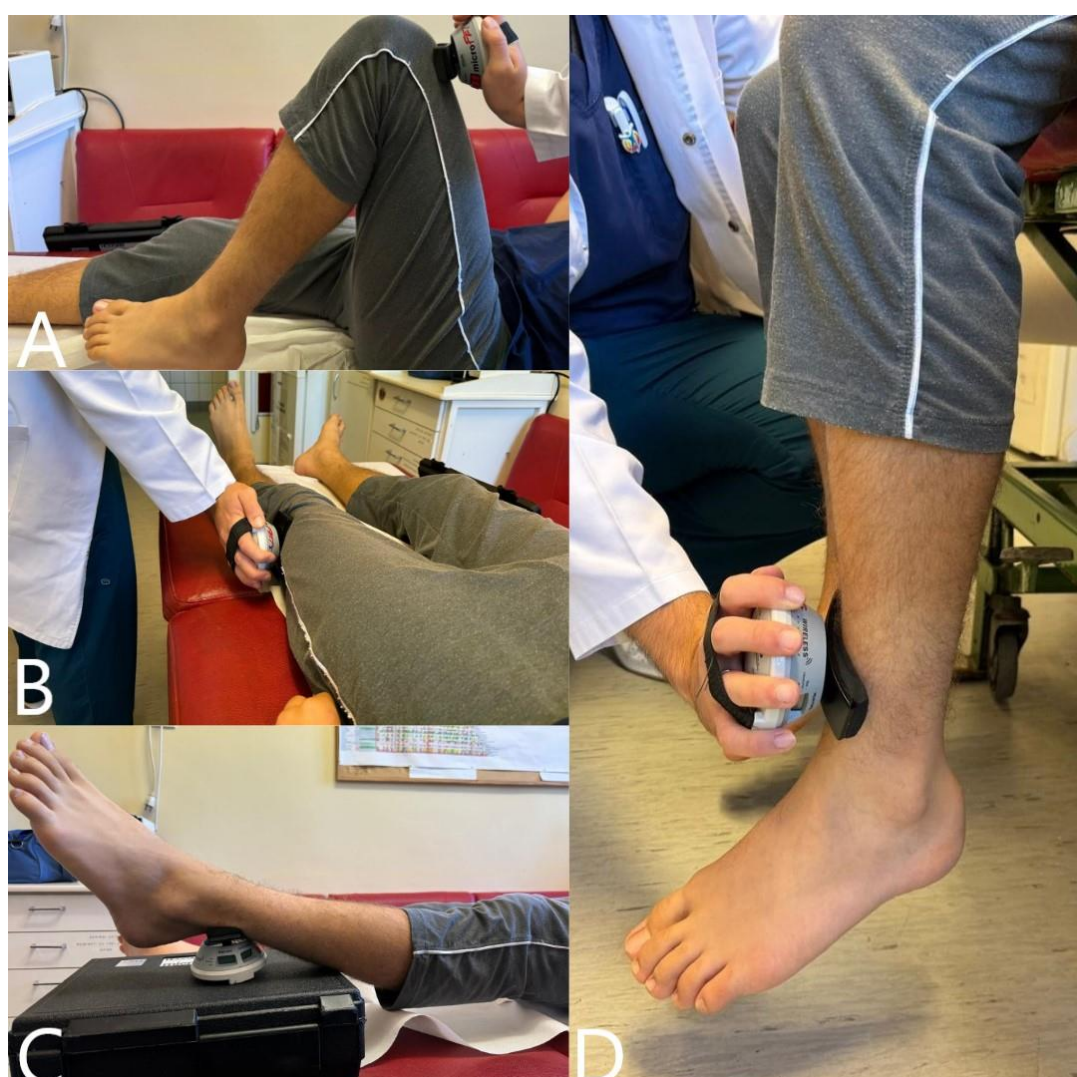
Isometric muscle force assessment: (**A**) left hip flexors, (**B**) left hip abductors, (**C**) left hip extensors, and (**D**) left knee extensors.

Measurements were performed for both the right and left lower limbs, in the following order: right hip flexors, left hip flexors (supine position with hip in 90° flexion, dynamometer proximal to the femoral condyles, Figure 5A); right hip abductors, left hip abductors (supine position and the opposite leg slightly flexed, dynamometer proximal to the lateral knee joint line); right hip adductors, left hip adductors (supine position and the opposite leg slightly flexed, dynamometer proximal to the medial knee joint line, Figure 5B); right hip extensors, left hip extensors (supine position, knee extended with the distal limb supported on a block, dynamometer just distal to malleoli on Achille’s tendon, Figure 5C); right knee extensors, left knee extensors (sitting, hip and knee in 90° flexion, dynamometer anterior on the leg, just proximal to malleoli, Figure 5D); right knee flexors, left knee flexors (sitting, hip and knee in 90° flexion, dynamometer just proximal to malleoli on Achille’s tendon).

The isometric muscle force (measured in Newton (N)) was recorded for 5–6 s. Each assessment was performed three times for each muscle group; the mean value was then calculated. The same examiner performed all of the muscle force determinations (M.N.).

### 2.3. Statistics

Statistics were performed using GraphPad Prism 5.0 for Windows [16]. Descriptive statistics were calculated for all variables (mean and standard deviation). Normal distribution of values was verified using the D’Agostino–Pearson normality test. Comparisons between the isometric muscle force of the right and left hips and knees were performed using Student’s unpaired *t*-test. Paired *t*-tests were used to compare the intragroup data (stabilometry parameters in the three different situations). A *p*-value less than 0.05 was considered statistically significant [16].

## 3. Results

Table 2 includes the stabilometric data in the three testing conditions. When comparing the eyes open and eyes closed situations, there were significant differences in CoP path length (*p* < 0.0001) and maximum CoP speed (*p* = 0.048), with higher values when tested with eyes closed. When comparing the eyes open and head retroflexed situations, the stabilometry parameters were not significantly different. When comparing the eyes closed and head retroflexed situations, there were significant differences in CoP path length (*p* = 0.025), 90% confidence ellipse area (*p* = 0.049), and maximum CoP speed (*p* = 0.023); the increased values were recorded when tested with eyes closed.

When comparing the isometric muscle force of the hip and knee, there were no significant differences between the right side (affected side) and the left one (non-affected side) (Table 3). However, the muscle force of all of the tested muscle groups of the hip (flexors, extensors, abductors, and adductors) and knee (flexors and extensors) was higher for the healthy lower limb.

## 4. Discussion

Our study examined postural balance in children with unilateral SCFE who followed a surgical intervention (in situ fixation with a single percutaneous screw). Postural balance was quantified using stabilometric parameters. In contrast with other studies, we also compared the stabilometry in different testing situations, namely with eyes open, eyes closed, and the head retroflexed. These conditions can have an impact on postural static activities.

The study by Angélico et al. [17] evaluated abductor muscle force in moderate and severe SCFE. They compared the results of osteotomy at the base of the femoral neck and osteoplasty with contralateral in situ epiphysiodesis for mild SCFE, contralateral, healthy hips in individuals with SCFE, and hips from healthy subjects. They found that at the final follow-up (ranging between 1 year and 3 years), the mean hip abductor torque at 60° and 120° was significantly reduced in hips after an osteotomy at the base of the neck when compared with the contralateral non-slip side and controls. The authors stated that one year was not sufficient to regain abduction strength following surgery; improvements in muscle force were recorded between one and two years after surgery [17]. In our study, we did not record significant differences in hip abductor muscle force between the operated and healthy hips. Our patients had a mean follow-up of 18.38 months. A possible explanation for the fact that the patients in our study did not present differences in hip and knee isometric muscle force could be that the surgical intervention is a less invasive one; it is one recommended in grade I and II SCFE (based on midland moderate slips).

All of the patients in our study followed a different surgical technique, namely in situ fixation with a single percutaneous screw. For “in situ” percutaneous screw fixation of SCFE, we placed the patient supine on the orthopedic table, with the lower limb in internal rotation so that the patella was facing upwards and the hip was in slight abduction. No further reduction efforts were made before and after positioning the patient. We used the imaging intensifier in order to obtain good intraoperative antero-posterior and lateral views of the femoral neck and head. Under fluoroscopic guidance, we inserted a guide wire (k-wire) in the center of the femoral head, from the anterior cortex of the femoral neck base. We measure the length of the k-wire inserted into the bone by placing a second k-wire against the femoral neck parallel to that in the femur and the difference in the exposed end of the k-wire was the length of the screw. After that, we drilled with a cannulated drill bit on the guide wire using fluoroscopy in order to detect the penetration of the guide wire in the joint or pelvis. For fixation, we used 6.5 mm or 7.0 mm cannulated screws. The screw was placed on the guide wire and inserted under fluoroscopy to its center head position; the final intraoperative x-rays were taken in standard and different oblique positions to detect any eventual penetration of the screw into the hip joint [18,19].

Besides the assessment of hip abductor function, we did not find any studies that have compared the hip and knee muscle forces between the affected and healthy side in patients with unilateral SCFE. In our study, there were no significant differences in hip flexors, extensors, and adductors between the surgically treated lower limb and the healthy one. The affected and non-affected knee extensors and flexors’ muscle forces were not significantly different.

The study by Hébert et al. [20] aimed to establish hand-held dynamometry maximal isometric muscle torque reference values for healthy children and adolescents (age range between 4 years and 2 months and 17 years) who are developing typically. The maximum isometric muscle strength of the hip flexors, extensors, and abductors and knee flexors and extensors was determined with a push–pull hand-held dynamometer; torque was expressed in Newton meter [20]. The participants of our study were between 11 and 17 years old. As the muscle force values of our subjects were determined in N, we could not perform a comparison between our patients and the healthy children and adolescents included in the investigation of Hébert et al. [20]. In our study, we aimed to analyze possible correlations between stabilometric parameters and isometric hip and knee muscle force.

In a study, Letafatkar et al. [21] showed that misalignment of the lower extremities can affect postural stability with impact on static and dynamic activities such as walking, running, and swimming. Postural control can be improved with different rehabilitation programs aimed at improving balance and strengthening lower extremity muscles [21].

An important role in the stabilometry of children with SCFE is the fact that these children are overweight and obese. In a study regarding the influence of body mass index on postural balance and muscle strength, Prasetiowati et al. [22] proved that obese children had a significantly larger center of pressure than healthy children. There were no significant differences in hip extensor muscle strength between obese children and those of normal weight. However, the study showed that obese children have decreased postural balance when compared to healthy children.

In our study, 14 (53.84%) children were over overweight and 2 (7.69%) were obese. In the recent study by Montaz et al. [23], obesity was identified as a risk factor associated with the development of contralateral SCFE after a unilateral SCFE in adolescents who needed surgical intervention. We recommend that our obese and overweight patients lose weight in order to prevent a contralateral SCFE.

The regain of muscle force in patients who have followed an orthopedic surgical intervention of the lower limb is important for postural stability, balance, and performance of both daily and recreational sports activities [24,25]. The clinical implications of our study are related to the importance of adequate muscle force of the affected lower limb for performing activities in different situations (with eyes closed or the head retroflexed) in a physically active population group (children aged between 11 and 17 years). Involvement in usual recreational sports activities with injury prevention is essential for children and adolescents who have undergone surgical intervention for unilateral SCFE. Fracture prevention is extremely important for both the affected and healthy limbs. As in upper limb fractures [26], the study of the laterality of trauma should be monitored in children with SCFE who are involved in individual or collective sport-related activities.

The absence of muscle force testing before the surgical intervention and a short time after surgery can be considered a limitation of our study. The relatively small number of patients and the imbalanced gender distribution (92.3% males) are also limitations. We have in view to increase the number of study patients and to have a postsurgical follow-up of at least 3 years. We intend to continue the study by including another sample of children with surgically treated SCFE, namely those that will follow a supervised physical exercise program. The analysis of the stabilometric data and hip and knee muscle force in two different samples (with and without a supervised physical exercise program) will help us to have a more detailed view of the improvements in functioning in children with SCFE treated via in situ fixation with one percutaneous screw. The medium- and long-term follow-up (through stabilometric assessment and muscle force testing) in children who followed the aforementioned surgical intervention and comparison with healthy subjects are opportunities for future research.

## 5. Conclusions

In children with unilateral SCFE who followed a surgical intervention, assessed at least 4 months after surgery, no differences in hip and knee muscle force were noted between the affected and non-affected lower limb. When comparing the stabilometry between the three different examination situations (with eyes open, eyes closed, and the head retroflexed) the highest values were reported when tested with eyes closed. Better postural stability is acquired when assessed with eyes open or with the head retroflexed in contrast with the eyes closed testing.

## Figures and Tables

**Table 1:** Patients’ features.

Variables	Patients (N = 26)
Age (years) (mean ± SD)	14.08 ± 2.63
Gender	
Male, N	24
Female, N	2
Height (cm) (mean ± SD)	154.2 ± 5.71
Weight (kg) (mean ± SD)	69.65 ± 12.23
BMI (kg/m^2^) (mean ± SD)	25.14 ± 3.88

SD: standard deviation; N: number of patients; BMI: body mass index.

**Table 2:** Stabilometric data in the testing conditions.

Stabilometric Data	Eyes Open	Eyes Closed	Head Retroflexed	*p* ^a^	*p* ^b^	*p* ^c^
CoP path length (mm) (mean ± SD)	359.8 ± 108.3	423.8 ± 158.2	374.8 ± 91.74	<0.0001 *	0.213	0.025 *
90% confidence ellipse area (mm^2^) (mean ± SD)	246.1 ± 155.4	248.2 ± 119.2	195.8 ± 113.7	0.933	0.133	0.049 *
Maximum CoP speed (mm/s) (mean ± SD)	95.46 ± 42.94	108.5 ± 43.17	90.77 ± 32.76	0.048 *	0.52	0.023 *

SD: standard deviation; CoP: center of pressure; *p* ^a^ relates to the difference between eyes open and eyes closed conditions; *p* ^b^ relates to the difference between eyes open and head retroflexed conditions; *p* ^c^ relates to the difference between eyes closed and head retroflexed conditions. * The differences are statistically significant.

**Table 3:** Comparisons of isometric hip and knee muscle force of the affected and non-affected side.

Muscle Force	Right Side (Affected Side)	Left Side (Non-Affected Side)	*p*
Hip flexors (N) (mean ± SD)	140.1 ± 44.46	154.1 ± 43.81	0.25
Hip extensors (N) (mean ± SD)	174.8 ± 76.29	195.3 ± 81.58	0.35
Hip abductors (N) (mean ± SD)	148.8 ± 66.76	174.2 ± 68.04	0.18
Hip adductors (N) (mean ± SD)	167.0 ± 67.5	191.0 ± 81.87	0.25
Knee flexors (N) (mean ± SD)	173.2 ± 95.36	199.7 ± 106.4	0.34
Knee extensors (N) (mean ± SD)	200.6 ± 89.48	231.8 ± 95.54	0.22

SD: standard deviation. When analyzing the correlations between the stabilometric parameters in the eyes open condition and isometric hip and knee muscle force (Table 4), we found significant negative correlations between the 90% confidence ellipse area and left knee extensors, right knee flexors, and left knee flexors, respectively. The higher the muscle force of knee flexors (affected and non-affected) and non-affected knee extensors is, the lower the confidence area. A significant negative correlation was also recorded between the maximum CoP speed and the left knee extensors’ muscle force; a higher non-affected knee extensors’ muscle force is related to a decreased CoP speed.

**Table 4:** Correlations between stabilometric parameters (eyes open condition) and isometric hip and knee muscle force.

	CoP Path Length		90% Confidence Ellipse Area		Maximum CoP Speed	
	R	*p*	r	*p*	r	*p*
Right hip flexors	0.13	0.49	0.29	0.14	0.82	0.68
Left hip flexors	0.058	0.77	0.21	0.30	−0.002	0.99
Right hip extensors	0.10	0.61	0.23	0.25	0.12	0.54
Left hip extensors	0.09	0.64	0.22	0.25	0.10	0.60
Right hip abductors	−0.04	0.84	0.10	0.61	0.048	0.81
Left hip abductors	0.092	0.65	0.34	0.08	0.21	0.28
Right hip adductors	0.031	0.87	0.27	0.17	0.13	0.49
Left hip adductors	−0.034	0.86	0.19	0.33	0.058	0.77
Right knee flexors	−0.15	0.43	−0.40	0.041 *	−0.19	0.34
Left knee flexors	−0.16	0.41	−0.45	0.02 *	−0.30	0.12
Right knee extensors	−0.047	0.81	−0.32	0.10	−0.23	0.25
Left knee extensors	−0.254	0.21	−0.43	0.028 *	−0.45	0.019 *

r: rank correlation coefficient. * The differences are statistically significant.

## Data Availability

The data presented in this study are available from the corresponding author (A.R.D.) upon request.

## References

[1] Aronsson D.D., Loder R.T., Breur G.J., Weinstein S.L. (2006). Slipped Capital Femoral Epiphysis: Current Concepts. J. Am. Acad. Orthop. Surg..

[2] Zusman N.L., Goldstein R.Y., Yoo J.U. (2024). Quantifying Risk Factors for Slipped Capital Femoral Epiphysis and Postslip Osteonecrosis. J. Pediatr. Orthop..

[3] Ionescu A., Dragomirescu M.-C., Herdea A., Ulici A. (2023). Developmental Dysplasia of the Hip: How Many Risk Factors Are Needed?. Child..

[4] Goodwin R.C., Mahar A., Wedemeyer M., Wenger D. (2007). Abductor Length Alterations in Hips with SCFE Deformity. Clin. Orthop. Relat. Res..

[5] Gao Y., Lyu X., Liu Q., Meng Y., Wang J., Pan S. (2021). Quantitative Evaluation of Hip Muscle Atrophy in Patients with Unilateral Slipped Capital Femoral Epiphysis Based on Magnetic Resonance Imaging. Acad. Radiol..

[6] Sangeux M., Passmore E., Gomez G., Balakumar J., Graham H.K. (2014). Slipped Capital Femoral Epiphysis, Fixation by Single Screw in Situ: A Kinematic and Radiographic Study. Clin. Biomech..

[7] Kapteyn T.S., Bles W., Njiokiktjien C.J., Kodde L., Massen C.H., Mol J.M. (1983). Standardization in Platform Stabilometry Being a Part of Posturography. Agressologie.

[8] Nagymate G., Kiss R. (2016). Replacing Redundant Stabilometry Parameters with Ratio and Maximum Deviation Parameters.

[9] Delfa-de la Morena J.M., Castro E.A., Rojo-Tirado M.Á., Bores-García D. (2021). Relation of Physical Activity Level to Postural Balance in Obese and Overweight Spanish Adult Males: A Cross-Sectional Study. Int. J. Env. Res. Public Health.

[10] Orofino F., Sgrò F., Coppola R., Crescimanno C., Lipoma M. (2015). Examining the Influence of Different Physical Activity Training on the Postural Stability of University Students. Int. J. Hum. Mov. Sport Sci..

[11] van Melick N., Meddeler B.M., Hoogeboom T.J., Nijhuis-van der Sanden M.W.G., van Cingel R.E.H. (2017). How to Determine Leg Dominance: The Agreement between Self-Reported and Observed Performance in Healthy Adults. PLoS ONE.

[12] Faul F., Erdfelder E., Buchner A., Lang A.-G. (2009). Statistical Power Analyses Using G*Power 3.1: Tests for Correlation and Regression Analyses. Behav. Res. Methods.

[13] PoDATA 3.0. https://www.chinesport.com/catalog/posture-analysis/podoscopes/03021-podata-3-0.

[14] Nagymáté G., Kiss R.M. (2016). Parameter Reduction in the Frequency Analysis of Center of Pressure in Stabilometry. Period. Polytech. Mech. Eng..

[15] Manual Muscle Tester | Digital Handheld Dynamometer | MicroFET2. Hoggan Scientific.

[16] Free Updates to Prism Windows 5.04 and Prism Mac 5.0f for Current Prism 5 Us-Ers. https://www.graphpad.com/support/prism-5-updates/.

[17] Angélico A.C.C., Garcia L.M., Icuma T.R., Herrero C.F., Maranho D.A. (2018). The Results of Osteotomy at the Base of Femoral Neck with Osteoplasty in Restoration of Abductor Function and Strength in Slipped Capital Femoral Epiphysis. Bone Jt. J..

[18] Flynn J.M., Wiesel S.W. (2010). Operative Techniques in Pediatric Orthopaedics.

[19] Ibrahim S. (2015). Tachdjian’s Pediatric Orthopaedics: From the Texas Scottish Rite Hospital for Children. Malays. Orthop. J..

[20] Hébert L.J., Maltais D.B., Lepage C., Saulnier J., Crête M. (2015). Hand-Held Dynamometry Isometric Torque Reference Values for Children and Adolescents. Pediatr. Phys. Ther..

[21] Letafatkar A., Zandi S., Khodaei M., Belali J. (2013). Flat Foot Deformity, Q Angle and Knee Pain Are Interrelated in Wrestlers. Nov. Physiother..

[22] Prasetiowati L., Kusumaningtyas S., Tamin T.Z. (2017). Effect of Body Mass Index on Postural Balance and Muscle Strength in Children Aged 8-10 Years. J. Krishna Inst. Med. Sci..

[23] Momtaz D., Mirghaderi P., Gonuguntla R., Singh A., Mittal M., Burbano A., Hosseinzadeh P. (2024). Rate and Risk Factors for Contralateral Slippage in Adolescents Treated for Slipped Capital Femoral Epiphysis: A Comprehensive Analysis of 3528 Cases. J. Bone Jt. Surg. Am..

[24] Petroman R., Rata A.L. (2020). Balance Performance in Sedentary and Active Healthy Young Individuals—A Cross-Sectional Study. Phys. Educ. Stud..

[25] Xhardo K., Iacob G., Cotrobas-Dascalu V.-T., Cordun M., Stoica M., Pelin F., Baltag O., Predescu C., Gherghel C., Bratu M. (2023). The Impact of the Biodex 4 pro System Dynamometer in the Rehabilitation of Ankle Sprain in Youth Football Players. Balneo PRM Res. J..

[26] Herdea A., Ulici A., Toma A., Voicu B., Charkaoui A. (2021). The Relationship between the Dominant Hand and the Occurrence of the Supracondylar Humerus Fracture in Pediatric Orthopedics. Children.

